# Usability and Utility of a Mobile App to Improve Medication Adherence Among Ambulatory Care Patients in Malaysia: Qualitative Study

**DOI:** 10.2196/15146

**Published:** 2020-01-31

**Authors:** Sara Chew, Pauline Siew Mei Lai, Chirk Jenn Ng

**Affiliations:** 1 Department of Primary Care Medicine University of Malaya Kuala Lumpur Malaysia

**Keywords:** medication adherence app, usability testing, utility testing

## Abstract

**Background:**

To date, several medication adherence apps have been developed. However, the existing apps have been developed without involving relevant stakeholders and were not subjected to mobile health app guidelines. In addition, the usability and utility of these apps have not been tested with end users.

**Objective:**

This study aimed to describe the usability and utility testing of a newly developed medication adherence app—Med Assist—among ambulatory care patients in Malaysia.

**Methods:**

The Med Assist app was developed based on the Theory of Planned Behavior and the Nielson usability model. Beta testing was conducted from March to May 2016 at a primary care clinic in Kuala Lumpur. Ambulatory care patients who scored ≥40% on the electronic health literacy scale, were aged ≥21 years, and were taking two or more long-term medications were recruited. Two rounds of in-depth interviews were conducted with each participant. The first interview, which was conducted upon participant recruitment, was to assess the usability of Med Assist. Participants were asked to download Med Assist on their phone and perform two tasks (register themselves on Med Assist and enter at least one medication). Participants were encouraged to “concurrently think aloud” when using Med Assist, while nonverbal cues were observed and recorded. The participants were then invited for a second interview (conducted ≥7 days after the first interview) to assess the utility of Med Assist after using the app for 1 week. This was done using “retrospective probing” based on a topic guide developed for utilities that could improve medication adherence.

**Results:**

Usability and utility testing was performed for the Med Assist app (version P4). A total of 13 participants were recruited (6 men, 7 women) for beta testing. Three themes emerged from the usability testing, while three themes emerged from the utility testing. From the usability testing, participants found Med Assist easy to use and user friendly, as they were able to complete the tasks given to them. However, the details required when adding a new medication were found to be confusing despite displaying information in a hierarchical order. Participants who were caregivers as well as patients found the multiple-user support and pill buddy utility useful. This suggests that Med Assist may improve the medication adherence of patients on multiple long-term medications.

**Conclusions:**

The usability and utility testing of Med Assist with end users made the app more patient centered in ambulatory care. From the usability testing, the overall design and layout of Med Assist were simple and user friendly enough for participants to navigate through the app and add a new medication. From the participants’ perspectives, Med Assist was a useful and reliable tool with the potential to improve medication adherence. In addition, utilities such as multiple user support and a medication refill reminder encouraged improved medication management.

## Introduction

### Background

Medication adherence is defined as the “extent to which a person’s behaviour-taking medication, following diet, and/or executing lifestyle changes corresponds with agreed recommendations from a healthcare provider” [1]. Unintentional nonadherence to medications is an important issue that needs to be addressed among ambulatory care patients, as they are responsible for the procurement and correct administration of their own medication(s) [2]. Nonadherence to medications not only affects patient clinical health outcome, but also patient safety, health care costs, and health care service utilization [1]. In the United States, 50%-71% of ambulatory care patients have two or more chronic diseases, which increases their risk of medication administration errors [3] such as taking unauthorized medications, taking an extra dose or the wrong dose, taking medications at the wrong time or frequency, or omitting a dose [4,5]. The prevalence of nonadherence to long-term medications in developed countries ranged from 50% to 70% [1,3], while in developing countries, the rate of nonadherence ranged from 40% to 80% [1,6,7]. Studies in Malaysia reported that 47%-53% of patients were nonadherent [8,9]. Most errors that occur in ambulatory care setting are preventable [10], and medication administration–related errors represent the largest component of preventable medication errors [11]. However, little is known about the extent of medication administration–related errors in the ambulatory care setting, as most studies were conducted in an inpatient setting [1,12,13].

Several interventions have been developed to improve medication adherence and reduce medication administration–related errors [14,15]. However, these interventions have varying success rates. These interventions usually involve a health care professional (doctor, pharmacist, or nurse) in preparing the patient’s pillbox, providing patient counselling and education [16,17], simplifying the medication regime [18], involving the patient in shared decision making [19], or improving patient-provider communication [14,15].

In recent years, mobile health (mHealth)—defined as “medical and public health practice supported by mobile devices, such as mobile phones, patient monitoring devices, personal digital assistants, and other wireless devices” [20]—has provided a new platform that could improve a patient’s medication adherence [21] (defined as “the extent to which a person’s behaviour- taking medication, following a diet, and/or executing lifestyle changes corresponds with the agreed recommendations from a health care provider”) [1]. Mobile apps have the potential to improve medication adherence and management of medications [22,23] through an active reminder system [24].

To date, several smartphone medication adherence apps have been developed [25]. However, existing apps were developed without involving relevant stakeholders (such as health care professionals or patients) [24,26] and were not subjected to mobile health app guidelines [23,27]. This may have resulted in some apps being “sub-standard” [27-29]. Some existing apps are also commercial in nature, requiring a subscription fee; are available only in one operating software platform; or can only be used in countries where it was developed [25]. Usability is defined as “the extent to which a product can be used by specified users to achieve specified goals with effectiveness, efficiency and satisfaction in a specified context of use” [30]. Utility is defined as the functionality of the app and how useful it is to users [31]. To the best of our knowledge, no study has assessed the usability and utility of a medication adherence app once it has been developed.

### Objectives

This study aimed to assess the usability and utility of a newly developed medication adherence app—*Med Assist*—among real end users.

## Methods

### Study Design

A qualitative methodology was utilized to obtain an in-depth understanding of end users’ experiences when using *Med Assist* from the usability and utility aspects.

### Beta Testing in the Development Phase

**Figure 1:**
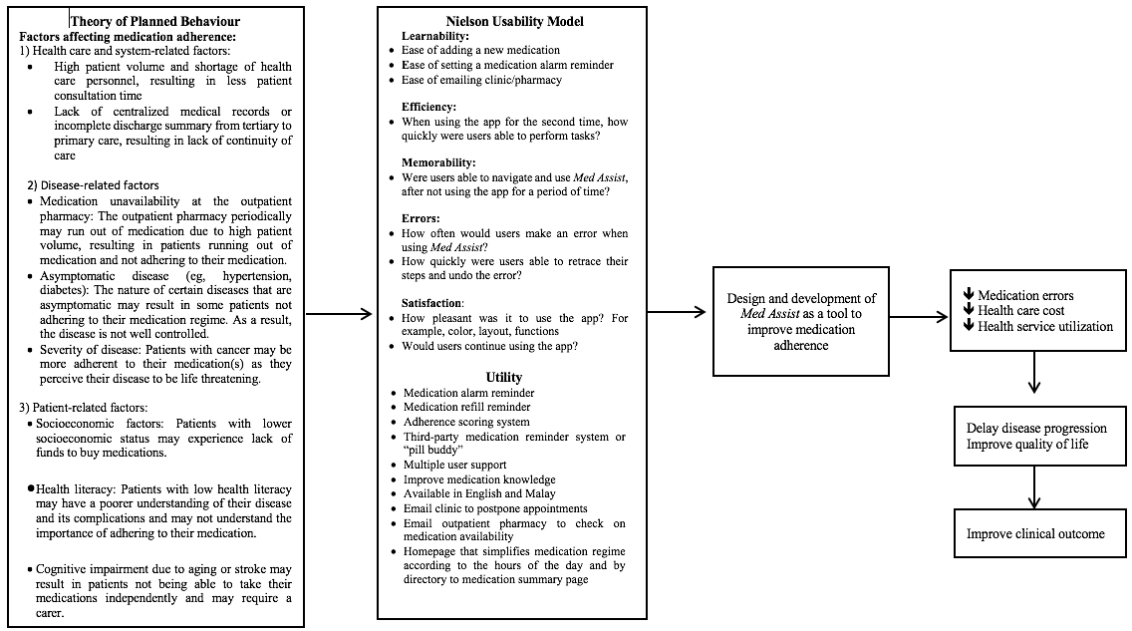
The conceptual framework for the design and development of Med Assist based on the Theory of Planned Behavior and the Nielson Usability Model.

The development phase consisted of two parts: alpha and beta testing. Alpha testing involved testing the design of *Med Assist* using a conceptual framework (Figure 1), which combined the Theory of Planned Behavior and the Nielson usability model and a utility model. To the best of our knowledge, existing frameworks on the design of mobile apps solely focused on the usability of the app. Most medication adherence apps did not incorporate health behavior theories [32]. We decided to develop our own framework, as we wanted to incorporate both the usability and utility of *Med Assist*. Additionally, our framework also included factors that could affect medication adherence. The design of Med Assist was also based on the utilities requested by the steering committee (Table 1).

**Table 1:** Summary of the preferred features and utilities of Med Assist.

Utility	Description
Specific medication reminder	Users will be able to set specific medication reminders with a personalized tone
Specific medication refill reminders	Users can set specific medication refill reminders, which prompt users to procure a prescription refill before running out of medications.
Complex medication regime	Ability to aid patients in managing complex medication regime.
Adherence scoring system	Users are able to calculate their adherence to medications. A 100% adherence to medications is displayed as five stars.
Multiple user support	Users can enter another individual’s list of medications in addition to their own.
Third party reminder or “pill buddy”	This function allows a family member to receive text message notification stating that the user has missed three consecutive medication reminders.
Contact clinic or pharmacy by email	Users can contact the clinic receptionist via email to postpone an appointment or the outpatient pharmacy to check on medication availability. This maximizes appointment schedules and allocates last-minute vacant slots to other patients.
Available in dual language	*Med Assist* is available in English and Malay to reach out to a wider group of users in Malaysia.

### Setting and Study Period

**Figure 2:**
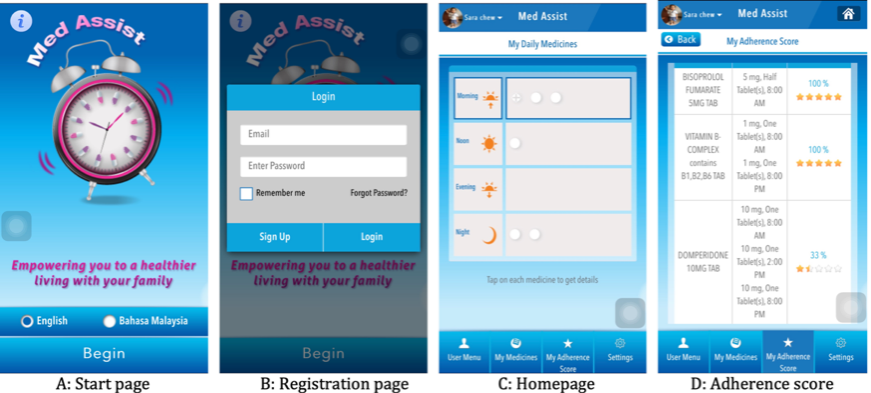
The start, registration, homepage, and adherence score of Med Assist (vP4).

**Figure 3:**
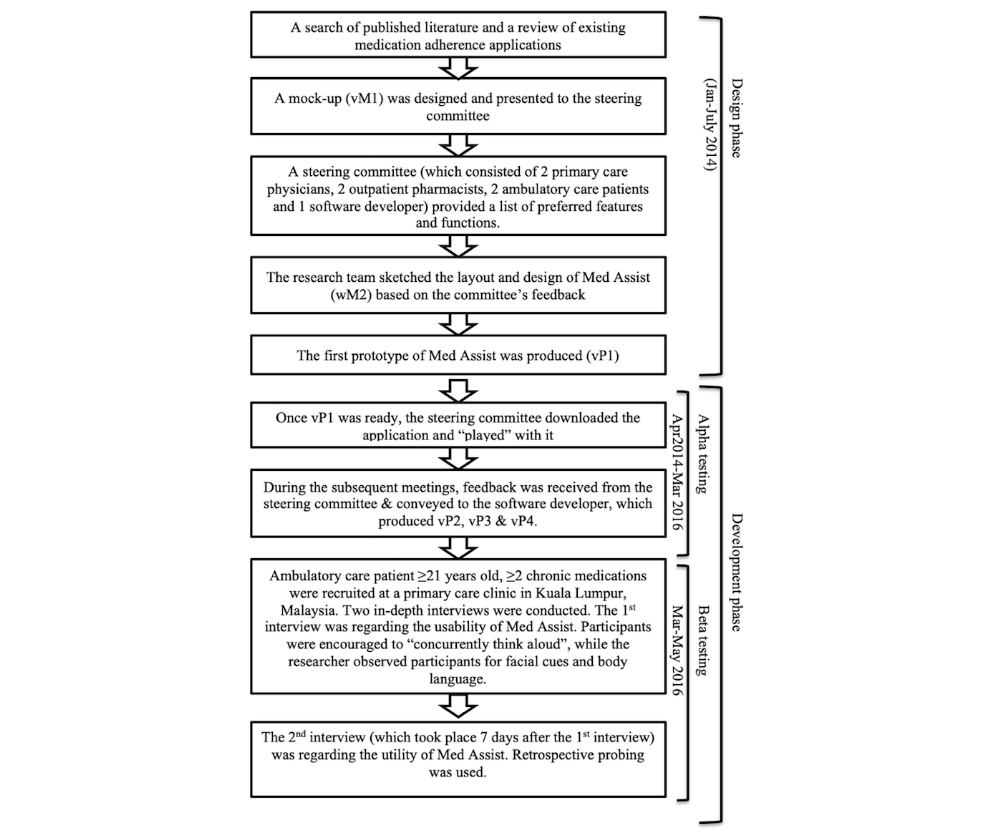
Flowchart of Med Assist design and development.

Med Assist vP4 (Figure 2) was the version used for beta testing. Beta testing was conducted from March to May 2016 (Figure 3). The first round of in-depth interviews was conducted at a primary care clinic in Kuala Lumpur, while the second round of in-depth interviews was conducted at a location convenient to the participants (eg, at their homes, a café nearby). In-depth interviews were conducted so that we could explore the views of participants regarding the usability and utility of Med Assist when using it for the first time. We asked participants to “concurrently think aloud,” so that their impressions and difficulties encountered could be recorded on the tape recorder. We also supplemented this interview process by observing the participant and documenting these observations as field notes. This allowed us to understand if they encountered any difficulties and whether they liked or disliked the utility. In-depth interviews allowed us to focus on the individual, assist the individual in using Med Assist, and create an environment where the individual would be able to express his/her views without being influenced by others.

### Participants

We included participants who had an electronic health literacy score of ≥40%, who understood English or Malay, and were taking two or more prescribed medications for their chronic condition. We excluded participants aged <21 years or who had mental disabilities. Purposive sampling was used to recruit older (≥65 years of age) and younger (<65 years of age) participants, as we wanted the experiences of older participants who may have more comorbidities, but may not be comfortable in using mobile apps, as well as younger participants who may have lesser comorbidities (than older persons) but may be more comfortable with using mobile apps. The purpose of recruiting participants based on age was to obtain a wider perspective when using *Med Assist*.

### Research Instruments

#### Topic Guide

A topic guide based on the Nielson usability model [33] and utility model was used to guide the in-depth interviews. This ensured that participants were asked for their experiences in terms of all aspects of user interface and user experience.

During the first interview, the usability of *Med Assist* was explored using the topic guide based on the Nielson usability model. This model was used, as it explored five components of usability: learnability (ease of navigating and using the app for the first time), efficiency (ease and speed of users navigating and using the apps for the second time), memorability (how much users remember regarding the use of the app after not using it for some time), errors (how often users would make an error using the app and how quickly they were able to retrace their steps and undo the error), and satisfaction (how pleasant the app was and if users continue would using it).

During the second interview, the utility of *Med Assist* was explored using a topic guide based on the utilities of *Med Assist* that would improve medication adherence and management, as decided by the steering committee during the design phase of *Med Assist*.

#### Med Assist Handbook

A *Med Assist* user manual (ie, a step-by-step guide on how to use *Med Assist*) was developed by the researcher and given to participants upon recruitment. This was to help participants explore *Med Assist* in their own time.

#### E-Health Literacy Scale

The electronic health literacy scale (eHEALS) was used to assess participants’ literacy skill in using their smart devices to find health-related information on the internet [34]. eHEALS, which consists of eight items, with a 4-point Likert scale, has been validated in Malaysia [34]. Participants who scored a higher eHEALS score indicated that they had higher literacy skills in using the internet as a resource to obtain more information.

### Ethics Approval

Ethics approval was obtained from the University of Malaya Medical Ethics Committee prior to the study (MECID no. 20143-12).

### Data Collection Process

A researcher approached participants who were “using” their smartphone while waiting to see their doctor. Participants were asked several “screening questions” such as “how often do you use your smartphone?” “what do you use your smartphone for?” “do you use any smartphone applications?” and “if yes, what applications do you use?” These questions were asked, so that the researcher could identify participants who used their smartphone as more than just a telecommunication device. For those who agreed to participate, written informed consent was obtained. Participants were then asked to fill in the demographic form and the eHEALS.

Each participant was interviewed twice. During the first interview, participants were encouraged to “concurrently think aloud” [35]. This method was employed so that the “first impression” of using *Med Assist* would be captured [35]. This enabled the researcher to capture any possible navigation issues that may arise from the end users’ perspective. The researcher took detailed notes and observed for nonverbal cues during each interview. Facial expressions and body language that were portrayed subconsciously by the participants were noted down by the researcher. Participants were encouraged to voice their first impressions and opinions about the app [36].

Participants were “probed retrospectively” during the second interview [35]. This was done by prompting participants with questions regarding the utilities of *Med Assist*. The researcher took detailed notes and observed for nonverbal cues. All interviews were audio recorded.

Participants were recruited until data saturation occurred. Data saturation was defined as “no new themes or codes emerging from interviews.” This was established when the next three participants recruited provided perspectives that were previously highlighted by other participants [37].

### Data Analysis

All interviews were transcribed verbatim. One researcher (SC) immersed herself in the data. An interpretive-descriptive approach was used to identify the themes that emerged from the data. This approach was used to obtain a deeper understanding of the usability and utility of *Med Assist* from the participants’ perspectives and experience of using the app. The researcher (SC) reflected on the data and began constructing an interpretive account of what the codes signified from the participants’ perspective and its application into clinical practice [38]. Four transcripts were first coded using the interpretive-descriptive approach that was based on the Nielson usability model and the utility model. This was done independently from the research team. SC also referred to the field notes for reflections, facial cues, and body language observed during the interviews. The research team then met to discuss the coding of the transcripts. Any coding discrepancies were resolved through discussion until a consensus was reached. Coding of the transcripts was performed using NVIVO v10 (QSL International Pty Ltd, Melbourne, Australia). Nodes were organized under larger categories, and the research team discussed the themes that emerged from the categories.

## Results

### Participants

A total of 13 participants (6 men, 7 women) were recruited (Table 2) for the first interview. Only 12 participants were interviewed consecutively (which occurred 2 weeks later), as one declined participation.

### Usability Testing

Three themes emerged from usability testing. They were challenges encountered when adding a new medication, with regard to patients’ understanding of their complex medication regime, and on the medication summary page.

#### Challenges Encountered When Adding a New Medication

Several subthemes emerged under this theme: confusion by terms used when adding medications into *Med Assist*, unfamiliarity with entering the generic name of the medication, and patients’ understanding of their complex medication regime.

**Table 2:** Demographic characteristics of participants recruited for beta testing.

ID^a^	Gender	Age (years)	Ethnicity	Level of education	Number of medication(s)	Patient/carer	iPhone/android user	eHEALS^b^ score (%)
P1	Male	66	Chinese	Secondary	3	Patient/carer	iPhone	84.4
P2	Female	29	Eurasian	Secondary	–^c^	Carer	Android	75.0
P3	Female	43	Chinese	Tertiary	4	Patient	Android	59.4
P4	Female	55	Indian	Secondary	2	Patient	Android	75.0
P5	Female	72	Malay	Tertiary	2	Patient	Android	75.0
P6	Male	56	Indian	Tertiary	3	Patient	iPhone	90.6
P7	Male	72	Malay	Secondary	4	Patient	Android	68.7
P8	Male	62	Chinese	Tertiary	6	Patient	Android	75.0
P9	Female	42	Malay	Tertiary	3	Patient	Android	46.9
P10	Female	64	Malay	Secondary	2	Patient/carer	Android	78.0
P11	Female	57	Indian	Tertiary	2	Patient	Android	50.0
P12	Female	27	Malay	Tertiary	4	Patient	Android	56.0
P13	Male	44	Malay	Tertiary	5	Patient	Android	75.0

^a^ID: identification.

^b^eHEALS: electronic health literacy scale.

^c^Not available.

#### Confusion by the Terms Used in Med Assist

Two terms—“timing” and “variable dosing”—confused most participants. In *Med Assist*, “timing” referred to the frequency with which users were required to take their medications (ie, daily or twice a day) rather than the time (ie, 7 AM) that they had to take their medications. Some participants understood what the term implied upon tapping on it.

Hmm..(Participant was initially confused by the term, but able to figure it out on her own) (taps on “timing) okay…once a day.. (participant thinks aloud)...

47 years old/female/android user/P3

How do I specify the frequency to take my medication? What does “timing” mean? What do I put here? (taps on timing) Oh I see!

72 years old/male/iOS user/P7

“Variable dosing” is a feature in *Med Assist* that allowed users to enter medications that are prescribed at different doses in one day; for example, 1700 mg metformin mane (taken in the morning) and 850 mg metformin nocte (taken at night). All participants were confused by this term, as most participants did not have medications with variable dosing. However, one participant who was prescribed 1700 mg metformin mane and 850 mg metformin nocte had no problems in entering metformin into *Med Assist* once the function of “variable dose” option was explained to him.

Okay...variable dose how...[taps on variable dose, switching it on]...850...take two tablets...no, strength is 850, strength supplied is 850 and strength to take is 850. I’m taking two tablets what...[after researcher helps him]...timing...twice a day, okay good...

56 years old/male/android user/P6

#### Unfamiliarity With the Generic Name of the Medication

When entering medication details into the app, most participants knew their medication by the brand name, but not by its generic name. *Med Assist* requires users to enter the generic name of the medication, as the pharmacy label only contains the generic name. Using generic names reduces the risk of confusion among patients, as different pharmacies may stock different brands of the same medication.

I’m not too sure what is the name of my medication. I know it by its brand name. I do know what the medication is for so having the indication automatically linked to the medication name is good to have.

72 years old/female/android user/P5

This part, I am not sure of the name of my medication, for example my cholesterol tablet, simvas or something.

47 years old/female/android user/P3

#### Patients’ Understanding of Their Complex Medication Regime

One participant was prescribed 75 μg levothyroxine daily but was supplied with 50 μg and 25 μg tablets. The participant was aware that she was supplied with two different strengths but was unaware that she had to enter the medication (levothyroxine) twice into *Med Assist,* treating 50 μg and 25 μg levothyroxine as two separate medications.

For strength prescribed it is 75 μg but I am given 50 μg and 25 μg by the pharmacy. Okay so for strength to take I should enter 75 μg because that is what I am taking, but the app won’t let me enter 75 μg. Why is that?

54 years old/female/android user/P4

Most participants understood their medication regime and the reason for taking their medication.

Yes I do know my medication regime better after using Med Assist. I have previously been relying on my wife who takes care of my medication supply and gives me my tablets in the morning.

44 years old/male/android user/P13

#### Medication Summary Page

Once all the details of the medication were entered, a summary page appears. The medication summary page could be accessed from the home page by tapping on the medication icon. All participants found this feature useful, as a list of all the medications that they had to take was summarized onto one page.

Oh having a medication summary page makes sense. This is very useful, makes the information simplified and more organized. It’s nice to have this feature.

64 years old/female/android user/P10

### Utility Testing

Two themes emerged from the utility testing of *Med Assist*. They were utilities that could improve medication adherence and the management of medication(s).

#### Utilities That Could Improve Medication Adherence

Three subthemes emerged: a medication alarm reminder system, an adherence scoring system, and the pill buddy option.

#### A Medication Alarm Reminder System

All participants found the medication alarm reminder useful, including the customizable alarm tone. One participant expressed concern that if the device was not with the user when the alarm rang, the alarm reminder would not have reminded the user to adhere to his/her medication regime. However, a snooze option was available on the medication reminder, which would remind users to take their medication at a later time. Another participant was unsure what time he/she could fix the reminder, as he/she did not have a fixed daily schedule.

Oh yes this was helpful. It prompted me to remind my mum to take her medications.

34 years old/female/android user/P2

Because I know… being ladies you tend to leave your handbag in the bedroom and you’re wandering around in the house and all that so I … the times that I actually took my phone with me, it does ring, it does ring that (short) tone beep. So if I were busy doing something else, I would have just missed it and of course it wouldn’t serve its purpose.

47 years old/female/android user/P3

Oh dear, the problem with setting the reminder is that I don’t have set times. It would have to depend on what time I wake up in the morning and go to bed in the evening, which varies day to day.

62 years old/male/android user/P8

#### A Medication Adherence Scoring System

Of the 13 participants, 7 were not aware that *Med Assist* could calculate their adherence score when they reported that they had taken their medication. However, one participant/caregiver who was adherent to her medications was shocked when her adherence score was zero. She then realized that *Med Assist* calculated her adherence score as 0, because she did not acknowledge that she had taken her medication by tapping on the medication reminder.

I’m sorry what was that? Oh! I wasn’t aware of that. So this would show what medications I have missed and when? And I am rated based on my adherence?

66 years old/male/iOS user/P1

Huh? I didn’t take my medicine? But...if I didn’t take my medicine, my husband also didn’t take the medicine? [This participant used the multiple user support feature] (After explanation) Oh that means each time I must tap on ‘take medicine.’ I didn’t know...”

64 years old/female/android user/P10

#### The Pill Buddy System

The pill buddy system allowed family members of a user to check that they had taken their medications remotely. Family members added as a “pill buddy” in the app will be sent a text message when the user missed taking their medication three times consecutively. Users who were caregivers found this feature useful, as they were able to “monitor” family members remotely.

Oh this is good. It allows me to keep an eye on my mum even when I am not with her.

34 years old/female/android user/P2

Oh I didn’t really explore that. I used the app for personal use only.

47 years old/female/android user/P3

#### Utilities to Improve the Management of Medications

Only one subtheme emerged: multiple user support system.

#### Multiple User Support

Participants who were not only patients but also caregivers were pleased to be able to enter medication details of the person under their care in the same app.

Oh I used this function to enter a separate profile for my mum’s medications. And it was amazing. It had all the tablet icons on the homepage and its own alarm reminder.

34 years old/female/android user/P2

## Discussion

### Principal Findings

From this study, three themes emerged from the usability testing of *Med Assist*, while another two themes emerged from the utility testing of *Med Assist*. *Med Assist* was designed and developed using the waterfall lifecycle software model. This model comprised five phases: users’ requirements, design, development of the software (*Med Assist* app), testing of the software app, and its release. The requirement phase was based on utilities that could improve medication adherence. This led to the design and development of *Med Assist* until the steering committee was satisfied with the prototype. The final prototype (version P4) was used for the beta testing of *Med Assist*. This paper focuses on the usability and utility (beta) testing of *Med Assist* where ambulatory care patients were recruited to use the app and provide feedback. *Med Assist* was designed for both iOS and android operating software systems. *Med Assist* was developed to be compatible with the technology (hardware and software) available in smart devices at the time of this study.

The steps to add a medication were simplified and displayed in a hierarchical order to prevent cognitive overload [39]. However, some participants struggled when adding a new medication due to the complexity of this task. Data input into a small device is challenging, as it requires the user to navigate through the app on a small screen [40]. In addition, the information displayed in a smaller screen device is crucial and needs to be in a hierarchical order [41]. This will ensure that users are able to process information without being overloaded or losing navigational direction when using the app [39,41]. Despite the challenges encountered by patients, the process of adding a new medication “manually” benefited patients with regard to their medication knowledge. Participants had to “learn” the generic name of their medications, administration frequency, and the purpose of their medication. In Malaysia, mandatory generic substitution is practiced by all public hospitals. Hospital outpatient pharmacies are required to only dispense generic medications regardless of the brand prescribed. At the time of this study, there were no published studies that reported the findings on medication data entry in an adherence app. Further research needs to be performed to fill this gap, as complex data entry can deter users from using the app [42].

The terms used in a medication adherence app should be self-explanatory. Although health care providers were involved in the design of *Med Assist*, the terms used in *Med Assist* confused participants. The term “timing” confused participants, as they thought that they had to enter the time that they took their medications. “Variable dosing” was added to offer flexibility to patients who were prescribed medications with variable dosing (eg, metformin). Individuals with lower literacy may require assistance when encountering such complex terms. This may lead to incorrect medication data entry, which compromises patient safety, leading to medication error and increased health care service utilization such as hospitalization and costs. One solution to improve the usability of *Med Assist* would be to have a “question mark icon” over the term, so that users could tap on the icon, which would then provide a brief explanation. The simplicity of the icon needs to be recognizable at a glance [43] and improve the user’s experience [40].

To our knowledge, no other study has reported the experiences of participants when using a medication adherence app. Participants also reported that the medication summary page, which was accessible through tapping on the medication icon on the homepage of *Med Assist* was useful, as it provided a concise overview of their medication details. This would further enhance the usability of *Med Assist*, as it would be more patient centered and more likely to be adopted.

Several studies have shown that behavioral change is achievable through active reminders, which reinforces the benefits of medication adherence apps [24,44]. A review in 2016 found that only 56% of medication adherence apps accommodate flexible scheduling for medication reminders [29]. Medication reminders with flexible scheduling (where users may opt for alternate days or weekly medication reminders) allows for personalization of the app to suit their individual needs. Participants also reported that they had a better understanding of the medication regime frequency and the indication of the medication, which appeared on the alarm reminder, thereby improving their medication knowledge. Improved patient knowledge is known to improve medication adherence and patient clinical health outcome [45]. However, areas involving strategies to improve patients’ medication knowledge requires further research [45,46]. Another useful utility was the multiple user support. Participants who were caregivers found this utility useful, as they were able to keep a separate profile and individualized a patient’s medication regime on the profile without affecting their own medication regime.

A review on mHealth apps found that most apps were based on a one-way reminder system (which sent reminders to users to take their medications) [32]. *Med Assist* offers a “snooze option,” which allowed users who were unable to take their medications at that point of time to take their medications later (ie, a two-way alarm reminder system). *Med Assist* required users to acknowledge the reminder that, theoretically, would make users more conscious of their adherence to their medications [32]. The medication alarm reminder actively prompted participants to take their medications correctly and on time. However, users who were aware of the adherence scoring system missed acknowledgement on the medication reminder. This resulted in their low adherence scores.

The adherence score serves as a gamification process (a process where the user is rewarded upon achieving the goal of the app) [47], which engaged users. Participants who knew about this feature were pleased that they were able to keep track of their adherence to their medication regime. A study on the design of an mHealth app for type 1 diabetes found that engaging users through a gamification process improved patient mediation adherence behavior [48].

In the Asian community, it is common for working adults to look after their elderly parents [49]. For younger individuals who still live with their elderly parents, the multiple user support function was a utility that enabled a separate profile to be created in addition to the patient’s own profile. Many working adults migrate to bigger cities to seek employment. Therefore, being present physically to ensure that their parents take their medications becomes difficult [50]. The pill buddy system allowed individuals to remotely check on their elderly parents’ medication adherence behavior. Although external monitoring utilities such as multiple user support and pill buddy system enable a third party to be more involved in patient care, a review in 2018 found that such functions were underutilized [46]. Future research could assess the external monitoring utilities, as they have the potential of improving patient medication adherence and its impact on patient clinical outcome.

### Strengths and Limitations

This study used a two-phase in-depth interview for the data collection process, which allowed us to explore patients’ and caregivers’ experience and perspectives in using the app for the first time and its utility after a period of using the app. To the best of our knowledge, no other medication adherence apps have conducted a similar study [46]. The usability and utility testing of *Med Assist* allowed us to understand the needs of ambulatory care patients and caregivers better and tailor *Med Assist* to suit their needs. This ensured that *Med Assist* would be a more patient-centered app and more likely to be used [31]. In addition, the wide demographic range of participants of this study provided experiences of both young and old users.

The generalizability of this study is limited by the use of a local hospital medication database. Although this simplified the entry of new medications into *Med Assist* (as a drop-down menu would appear and link to the indication of the medication), it limited the use of *Med Assist* to the local health care setting. There were several utilities of *Med Assist* that were not explored due to the short period between the two-phased in-depth interviews, such as medication refill reminders and the option to email health care professionals (ie, doctor, pharmacist). Future studies should have a longer time frame between the usability and utility testing to fully explore end users’ perspectives and experiences when using a medication adherence app. All participants recruited were from Kuala Lumpur (ie, a major city in Malaysia). A wider participant recruitment process should be carried out to explore the experiences and perspectives of rural end users. Due to time constraints, we were unable to incorporate the perspectives of the users in beta testing to modify *Med Assist* and retest *Med Assist* before progressing to the release phase.

### Conclusions

Our study found that the overall design and layout of *Med Assist* was simple and user friendly enough for participants to navigate and complete certain tasks. However, the process of adding a new medication was confusing for some participants and may require assistance features; The flexible medication alarm reminder and pill buddy system encouraged a positive change in patients’ medication adherence behavior. In addition, participants reported that the multiple user support and medication refill reminder encouraged better medication management. Our study suggests that *Med Assist* could aid ambulatory patients who are on long-term medications to improve medication adherence through active reminders.

## Abbreviations

eHEALS: electronic health literacy scale
mHealth: mobile health

## References

[1] (2003). Adherence to Long Term Therapiesvidence for Actions.

[2] (2016). Patient Safety in Ambulatory Settings.

[3] Schoen C, Osborn R, How SK, Doty MM, Peugh J (2008). In Chronic Condition: Experiences Of Patients With Complex Health Care Needs, In Eight Countries, 2008. Health Affairs.

[4] Barker KN, Flynn EA, Pepper GA, Bates DW, Mikeal RL (2002). Medication Errors Observed in 36 Health Care Facilities. Arch Intern Med.

[5] Schwartz D, Wang M, Zeitz L, Goss MEW (1962). Medication Errors Made by Elderly, Chronically Ill Patients. Am J Public Health Nations Health.

[6] Medi R, Mateti U, Kanduri K, Konda S (2018). Medication adherence and determinants of non-adherence among south Indian diabetes patients. Journal of Social Health and Diabetes.

[7] Thinking outside the pillbox: a system-wide approach to improving patient medication adherence for chronic disease.

[8] Paraidathathu T, Nur Sufiza A, Azuana (2012). Medication adherence among hypertensive patients of primary health clinics in Malaysia. PPA.

[9] Paraidathathu T, Islahudin F, Azuana, Ahmad (2013). Medication adherence in patients with type 2 diabetes mellitus treated at primary health clinics in Malaysia. PPA.

[10] Khoo EM, Lee WK, Sararaks S, Abdul Samad A, Liew SM, Cheong AT, Ibrahim MY, Su SHC, Mohd Hanafiah AN, Maskon K, Ismail R, Hamid MA (2012). Medical errors in primary care clinics--a cross sectional study. BMC Fam Pract.

[11] Cohen MM (2005). Medication safety program reduces adverse drug events in a community hospital. Quality and Safety in Health Care.

[12] Gandhi TK, Weingart SN, Borus J, Seger AC, Peterson J, Burdick E, Seger DL, Shu K, Federico F, Leape LL, Bates DW (2003). Adverse Drug Events in Ambulatory Care. N Engl J Med.

[13] Gandhi TK, Lee TH (2010). Patient Safety beyond the Hospital. N Engl J Med.

[14] Cross A, Elliott R, George J (2016). Interventions for improving medication-taking ability and adherence in older adults prescribed multiple medications. Cochrane Database of Systematic Reviews.

[15] Graves M, Roberts Michael C, Rapoff Michael, Boyer Amanda (2010). The efficacy of adherence interventions for chronically ill children: a meta-analytic review. J Pediatr Psychol.

[16] Powell LH, Calvin JE, Richardson D, Janssen I, Mendes de Leon CF, Flynn KJ, Grady KL, Rucker-Whitaker CS, Eaton C, Avery E, HART Investigators FT (2010). Self-management Counseling in Patients With Heart Failure. JAMA.

[17] Wu J, Corley DJ, Lennie TA, Moser DK (2012). Effect of a medication-taking behavior feedback theory-based intervention on outcomes in patients with heart failure. J Card Fail.

[18] Sadik A, Yousif M, McElnay JC (2005). Pharmaceutical care of patients with heart failure. Br J Clin Pharmacol.

[19] Solomon DH, Gleeson T, Iversen M, Avorn J, Brookhart MA, Lii J, Losina E, May F, Patrick A, Shrank WH, Katz JN (2009). A blinded randomized controlled trial of motivational interviewing to improve adherence with osteoporosis medications: design of the OPTIMA trial. Osteoporos Int.

[20] (2011). mHealth: New horizons for health through mobile technologies.

[21] nstitute of Medicine (US) Committee on Quality of Health Care in America (2001). Crossing the Quality Chasm: A New Health System for the 21st Century.

[22] Kaushal R, Bates D (2002). Information technology and medication safety: what is the benefit? Qual Saf Health Care. Qual Saf Health Care.

[23] (2013). U.S. Food and Drug Administration.

[24] Dayer LE, Shilling R, Van Valkenburg M, Martin BC, Gubbins PO, Hadden K, Heldenbrand S (2017). Assessing the Medication Adherence App Marketplace From the Health Professional and Consumer Vantage Points. JMIR Mhealth Uhealth.

[25] Dayer L, Heldenbrand S, Anderson P, Gubbins PO, Martin BC (2013). Smartphone medication adherence apps: Potential benefits to patients and providers: Response to Aungst. J Am Pharm Assoc.

[26] Heldenbrand S, Martin BC, Gubbins PO, Hadden K, Renna C, Shilling R, Dayer L (2016). Assessment of medication adherence app features, functionality, and health literacy level and the creation of a searchable Web-based adherence app resource for health care professionals and patients. J Am Pharm Assoc.

[27] U.S. Department of Health and Human Services Food and Drug Administration, Center for Devices and Radiological Health, Center for Biologics Evaluation and Research (2015). Mobile Medical Applications: Guidance for Food and Drug Administration Staff.

[28] Liu C, Zhu Q, Holroyd KA, Seng EK (2011). Status and trends of mobile-health applications for iOS devices: A developer's perspective. Journal of Systems and Software.

[29] Santo K, Richtering SS, Chalmers J, Thiagalingam A, Chow CK, Redfern J (2016). Mobile Phone Apps to Improve Medication Adherence: A Systematic Stepwise Process to Identify High-Quality Apps. JMIR Mhealth Uhealth.

[30] (1998). ISO 9241-11:1998(en) Ergonomic requirements for office work with visual display terminals (VDTs) — Part 11: Guidance on usability.

[31] (2003). Nielsen Norman Group logoNielsen Norman Group.

[32] Riley WT, Rivera DE, Atienza AA, Nilsen W, Allison SM, Mermelstein R (2011). Health behavior models in the age of mobile interventions: are our theories up to the task?. Transl Behav Med.

[33] Nielsen J (1993). Usability Engineering.

[34] Abdullah A, Mohd PN (2106). Internet use and eHealth literacy levels among patients attending a hospital based primary care clinic in Malaysia.

[35] Willis GB, Artino AR (2013). What Do Our Respondents Think We're Asking? Using Cognitive Interviewing to Improve Medical Education Surveys. Journal of Graduate Medical Education.

[36] Mason J (2002). Qualitative Researching. 2nd ed.

[37] Guest G, Bunce A, Johnson L (2016). How Many Interviews Are Enough?. Field Methods.

[38] Thorne S, Kirkham SR, O'Flynn-Magee K (2016). The Analytic Challenge in Interpretive Description. International Journal of Qualitative Methods.

[39] Mayer RE, Moreno R (2003). Nine Ways to Reduce Cognitive Load in Multimedia Learning. Educational Psychologist.

[40] Zhang D, Adipat B (2005). Challenges, Methodologies, and Issues in the Usability Testing of Mobile Applications. International Journal of Human-Computer Interaction.

[41] Broderick J, Devine T, Langhans E, Lemerise A, Lier S, Harris L (2014). Designing health literacy mobile apps.

[42] Teo CH, Ng CJ, White A (2017). What Do Men Want from a Health Screening Mobile App? A Qualitative Study. PLoS ONE.

[43] Harley A Nielsen Norman Group logoNielsen Norman Group.

[44] Vervloet M, Linn AJ, van Weert JCM, de Bakker DH, Bouvy ML, van Dijk L (2012). The effectiveness of interventions using electronic reminders to improve adherence to chronic medication: a systematic review of the literature. J Am Med Inform Assoc.

[45] Stinson JN, Jibb LA, Nguyen C, Nathan PC, Maloney AM, Dupuis LL, Gerstle JT, Alman B, Hopyan S, Strahlendorf C, Portwine C, Johnston DL, Orr M (2013). Development and Testing of a Multidimensional iPhone Pain Assessment Application for Adolescents with Cancer. J Med Internet Res.

[46] Ahmed I, Ahmad NS, Ali S, Ali S, George A, Saleem Danish H, Uppal E, Soo J, Mobasheri MH, King D, Cox B, Darzi A (2018). Medication Adherence Apps: Review and Content Analysis. JMIR Mhealth Uhealth.

[47] Deterding S, Dixon D, Khaled R, Nacke L (2011). From game design elements to gamefulness: defining.

[48] Cafazzo JA, Casselman M, Katzman DK, Palmert MR (2012). 133. Bant: An mHealth App for Adolescent Type I Diabetes – A Pilot Study. Journal of Adolescent Health.

[49] Ambigga KS, Ramli A, Suthahar A, Tauhid N, Clearihan L, Browning C (2011). Bridging the gap in ageing: Translating policies into practice in Malaysian Primary Care. Asia Pac Fam Med.

[50] Sim O (2002). Ageing in Malaysia: A review of national policies and programmes. Aging and Long Term Care: National Policies in the Asia-Pacific ISEAS.

